# Positive moods are all alike? Differential affect amplification effects of ‘elated’ versus ‘calm’ mental imagery in young adults reporting hypomanic-like experiences

**DOI:** 10.1038/s41398-022-02213-4

**Published:** 2022-10-19

**Authors:** Caterina Vannucci, Michael B. Bonsall, Martina Di Simplicio, Aimee Cairns, Emily A. Holmes, Stephanie Burnett Heyes

**Affiliations:** 1 School of Psychology and Educational Sciences, University of Bologna, Bologna, Italy; 2 School of Psychology, University of Birmingham, Birmingham, UK; 3 MoMiLab Research Unit, IMT School for Advanced Studies, Lucca, Italy; 4 Department of Biology, University of Oxford, Oxford, UK; 5 Division of Psychiatry, Department of Brain Sciences, Imperial College London, London, UK; 6 Warwick Medical School, University of Warwick, Coventry, UK; 7 Department of Psychology, Uppsala University, Uppsala, Sweden

**Keywords:** Human behaviour, Bipolar disorder

## Abstract

Positive mood amplification is a hallmark of the bipolar disorder spectrum (BPDS). We need better understanding of cognitive mechanisms contributing to such elevated mood. Generation of vivid, emotionally compelling mental imagery is proposed to act as an ‘emotional amplifier’ in BPDS. We used a positive mental imagery generation paradigm to manipulate affect in a subclinical BPDS-relevant sample reporting high (*n* = 31) vs. low (*n* = 30) hypomanic-like experiences on the Mood Disorder Questionnaire (MDQ). Participants were randomized to an ‘elated’ or ‘calm’ mental imagery condition, rating their momentary affect four times across the experimental session. We hypothesized greater affect increase in the high (vs. low) MDQ group assigned to the elated (vs. calm) imagery generation condition. We further hypothesized that affect increase in the high MDQ group would be particularly apparent in the types of affect typically associated with (hypo)mania, i.e., suggestive of high activity levels. Mixed model and time-series analysis showed that for the high MDQ group, affect increased steeply and in a sustained manner over time in the ‘elated’ imagery condition, and more shallowly in ‘calm’. The low-MDQ group did not show this amplification effect. Analysis of affect clusters showed high-MDQ mood amplification in the ‘elated’ imagery condition was most pronounced for *active* affective states. This experimental model of BPDS-relevant mood amplification shows evidence that positive mental imagery drives changes in affect in the high MDQ group in a targeted manner. Findings inform cognitive mechanisms of mood amplification, and spotlight prevention strategies targeting elated imagery, while potentially retaining calm imagery to preserve adaptive positive emotionality.

## Introduction

Positive mood amplification which can, at times, escalate rapidly and be maladaptive is a hallmark of the bipolar disorder spectrum (BPDS). BPDS is characterized by disabling mood states reflected in manic or hypomanic episodes (e.g., elevated, expansive, or irritable mood, and hyperactivity; [1, 2]), depressive episodes (e.g., low mood or loss of interest or pleasure), as well as mixed mood episodes [3, 4] and/or chronic affective instability [5]. BPDS is associated with high rates of disability [6], medical comorbidities [7–9] and suicidality [10]. Hypomania is a sub-manic state characterized by elevated and sometimes irritable mood, and can be measured along a continuum of experiences using self-report questionnaires [11]. The presence of high levels of self-reported hypomanic-like experiences is associated with risk for developing bipolar disorder [12]. Critically, we lack early or preventative *psychosocial* interventions specifically able to target hypomanic mood escalation. This is problematic as anti-manic pharmacological agents may not be favoured by young people at risk, due to their potential for side effects. We need better understanding of the cognitive mechanisms underlying hypomanic-like mood symptoms across the clinical and subclinical BPDS, in order to develop better psychological prevention and treatment strategies [13].

The current study focuses on the hallmark process leading to elevated mood, termed positive mood amplification. While positive affective states are often beneficial and appropriate, dysregulated positive mood is a key feature of (hypo)mania and BPDS [14, 15] comprising frequent, intense, long-lasting and context-insensitive positive affect and heightened responses to positive stimuli [16–21]. Elevated mood is interlinked with risk-taking, reduced sleep and socially inappropriate behaviour in BPDS, the so-called ‘dark side’ of positive emotion [19, 22, 23]. While the extreme facets of mania are targeted pharmacologically, there is a need for psychological interventions to address earlier positive mood escalation at preventative stages, especially in young people at risk of developing BPDS [12, 24]. However, on the flipside, positive affective experiences are centrally important for quality of life, and the desire to retain positive emotionality may undermine treatment compliance in BPDS [25]. We need better cognitive-mechanistic understanding of the boundary between benign versus maladaptive positive mood amplification, and the timescales on which this can operate. Such understanding could promote psychological interventions that reduce potentially harmful positive mood, while preserving aspects that are benign and indeed beneficial for quality of life. To this end, the current experimental study investigated two distinct drivers of positive mood amplification in a subclinical BPDS-relevant sample.

One cognitive mechanism hypothesised to drive mood amplification in BPDS is mental imagery [26]. Mental imagery is defined as the experience of perception in the absence of eliciting sensory input [27]; mental imagery of past, present, future, or fantasy events triggers affective processing in a manner like perception. Various attributes of mental imagery, such as the tendency to use imagery in daily life [28], as well as vividness [29] and emotional impact [30], have been shown to vary between individuals.

Mental imagery has been identified as a potential transdiagnostic risk mechanism and treatment target in a number of mental disorders [31–37]. Consequently, mental imagery paradigms are a potent experimental tool for manipulating affect (e.g., picture-word cue imagery generation paradigm; [38]). In BPDS, mental imagery is hypothesized to drive pathological mood amplification, exacerbating both manic and depressed states (Emotional Amplifier Theory; [26, 39]). Correlational and experimental evidence indicates greater tendency to experience mental imagery across the clinical and sub-clinical BPDS, and greater emotional impact of this imagery [30, 26, 40–44]. The current study, therefore, used an experimental mental imagery paradigm adapted from a prior study [45] to manipulate affect in a subclinical BPDS-relevant sample.

In our previous experimental study, a subclinical young adult sample reporting high levels of hypomanic-like experiences showed greater changes in self-reported affect in response to (i.e., pre/post) a computerized positive mental imagery generation task, compared to controls [45]. This evidence suggests that mental imagery drives short-term changes in affect in a BPDS-relevant sample in a manner congruent with mood amplification. However, a number of questions remain unaddressed. First, how specific is this effect to the eliciting conditions? Is BPDS-relevant positive mood amplification best characterized as a non-specific response across categories of affective stimuli (cf. [15]), or can the degree of amplification differ depending on the eliciting stimulus (cf. [46–48])? Second, how specific is the effect in terms of affective response? Is BPDS-relevant positive mood amplification characterized by non-specific amplification across affect categories? Alternatively, is amplification related to particular categories of affect, namely goal-directed positive affect (related to approach behavior and typically associated with (hypo)mania; [48]; in contrast to consummatory positive affect), or does it also apply to negative affective states that can additionally characterize hypomania and mixed states [45, 49]?

To address the first question (stimulus specificity), we sought to compare changes in self-reported affect across two eliciting stimulus categories, comprising ‘elated’ vs. ‘calm’ mental imagery generation conditions. Both experimental conditions consisted of positive stimuli; in the elated condition, stimuli featured reward-pursuit, ambitious achievements and competitive scenarios [48] while in the calm condition, stimuli depicted scenarios characterized by peace/contentment, rest, and self-acceptance/belonging [50]. To address the second question (response specificity) we investigated task-dependent changes in self-reported affect across distinct affect clusters, e.g., negative affect, positive affect associated with approach behaviour and excitement, and positive affect associated with calmness and contentment. Both stimulus specificity and response specificity hypotheses reflect distinctions between positive affect as approach motivation, and positive affect as consummatory and reflective subjective states [51]. To map the temporal profile of hypothesised mood change, we investigated affect change at a micro-level, i.e., within the experimental session, by eliciting self-reported affect ratings at four time-points: before, twice during, and after the imagery task.

Participants comprised a non-clinical community sample of young adults reporting either low or high levels of hypomanic-like experiences [52]. Adopting this spectrum approach takes into account the wide variability of symptoms at the subclinical level while remaining unconfounded by acute illness or medication state [53–55]. Hence, studying a subclinical population on the BPDS can lead to important insights on aetiology and treatment of bipolar disorder [15].

The aim of our study was to identify the impact of specific categories of positive mental imagery stimuli (‘elated’ vs. ‘calm’) on bipolar-relevant mood amplification, including across distinct categories of affective experience (positive-active, positive-calm, negative), since this may be informative for specific risk and treatment mechanisms. We had the following hypotheses and predictions. First, we predicted greater increases in affect following elated (vs. calm) imagery in participants reporting high levels of hypomanic-like experiences (Hypothesis 1: stimulus-specificity). Second, we predicted amplification related to particular affective clusters (Hypothesis 2: affect-specificity): increased positive (vs. negative) affect; and increased active, goal-directed (vs. calm, consummatory) positive affect. We predicted a moderating effect of imagery vividness on mood amplification, in line with prior studies [45]. We tested these hypotheses by comparing self-reported affect across groups, conditions, and time-points of a positive imagery generation task.

## Method

### Participants

The sample consisted of 61 adults (45 women, 13 men, 1 other) aged 18–25 (M = 20.53, SD = 1.8; see Table 1). Participants were recruited through posters and online advertisements on social media and specific websites at the University of Birmingham and in the local community. The study was approved by the University of Birmingham Science, Technology, Engineering, and Mathematics Ethical Review Committee (ERN_15-1435). Participants gave their written, informed consent at pre-screening and again before the psychiatric screening and experimental session. After completion of the session, participants were debriefed and received compensation for their participation (£10/hour).

**Table 1:** Demographic characteristics, emotional measures, and general imagery measure for high and low MDQ.

Characteristics	Low MDQ (*n* = 30)		High MDQ (*n* = 31)	
	M	SD	M	SD
Age (years)	20.63	1.93	20.42	1.68
SUIS	39.27	7.46	42.16	7.07
BDI-II	6.93	4.43	7.57	6.20
STAI-T	39.57	9.79	44.27	13.03
ALS-SF	1.73	0.44	1.79	0.54
ACS*	2.92	0.71	3.45	0.84
AIM*	3.49	0.47	3.78	0.50
Gender (#/%)				
Female	22 (73.3%)		25 (80.6%)	
Male	8 (26.7%)		5 (16.1%)	
Other	0 (0%)		1 (3.2%)	
Occupation (#/%)				
Student	28 (93.3%)		30 (96.8%)	
Non-student	2 (6.7%)		1 (3.2%)	
Ethnicity (#/%)				
White	24 (80.0%)		22 (71%)	
Other	6 (20%)		9 (29%)	
DSM-5 Disorder (#/%)				
Lifetime	12 (38.7%)		19 (61.3%)	
Anxiety	6 (40%)		9 (60%)	
Substance Use	3 (42.9%)		4 (57.1%)	
Depressive Episode	10 (37%)		17 (63%)	

*SUIS* Spontaneous Use of Imagery Scale, *BDI-II* Beck Depression Inventory-II, *STAI-T* Spielberger State-Trait Anxiety Inventory, *ALS* Affective Lability Scales- Short form, *ACS* Affective Control Scale, *AIM* Affective Intensity Measure, *DSM-5 Disorder* Mini psychiatric diagnosis. *Group difference significant at *P* < 0.5

#### Participant pre-screening and exclusion criteria

To recruit individuals across a spectrum of hypomanic-like experiences, *N* = 255 young adults were pre-screened online by completing section A of the Mood Disorder Questionnaire (MDQ; [52]). Participants were categorized according to the number of hypomanic experiences reported on the MDQ section A (0–13): high (≥7; range = 7–13), medium (range = 4–6), or low (≤3; range = 0–3). Participants categorized as high or low on the MDQ were potentially eligible to attend the experimental session. We further implemented screening based on the Spontaneous Use of Imagery Scale (SUIS; [56]) to exclude participants with a particularly low tendency to use imagery spontaneously (SUIS score of 23 or less), and who therefore might not be able to perform the experimental task. Our screening resulted in exclusion of 52 participants scoring medium on the MDQ and 3 scoring ≤23 on the SUIS. Subsequently, two eligible participants indicated they were no longer interested in participating. From the remaining sample, high (*n* = 45) and low (*n* = 31) MDQ scoring participants were contacted to attend psychiatric screening using the Mini International Neuro-psychiatric Interview for DSM-5 (MINI; [57]) (see Supplementary Material). Exclusion criteria (resulting in exclusion of 11 participants) included: (hypo)manic (current and past), depressive and psychotic episodes (current; for full exclusion criteria see Supplementary Material). One participant was excluded due to faulty administration of the psychiatric screening [57]. Finally, we excluded 4 participants based on poor comprehension of the imagery generation task instructions. The final sample of *N* = 61 consisted of *n* = 31 participants scoring high on the MDQ and *n* = 30 with a low MDQ score.

### Procedure

**Fig. 1:**
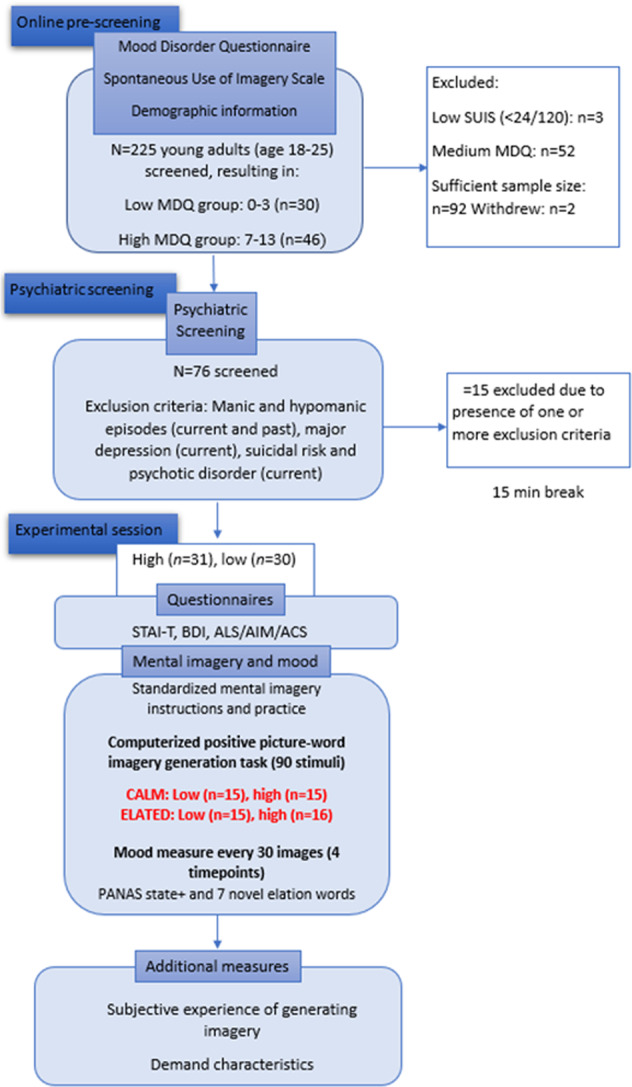
Study procedure. Online pre-screening; in-person psychiatric screening using the MINI; experimental session consisting of pre/post questionnaires, standardized imagery generation training procedure, and elated or calm positive picture-word cue imagery generation task with in-task affect rating.

Demographic characteristics were collected at online pre-screening via LimeSurvey. Following the psychiatric screening and a 15-minute break, eligible participants completed self-report baseline affect questionnaires, a self-report measure of current mood, and valence ratings of picture pleasantness (see Measures and Supplementary Material). Participants were then randomised to one of two imagery conditions and in both cases received a standardized imagery generation training procedure followed by the elated or calm positive picture-word cue imagery generation task (see Measures). Subsequently, participants repeated the valence ratings of picture pleasantness task and gave feedback on the imagery generation task (see Measures). Finally, participants were debriefed, thanked, and compensated. See Fig. 1.

### Measures

#### Demographic, pre-screening, and baseline affect questionnaires

See Supplementary Material.

#### Mental imagery training

Participants completed a standardised imagery generation training procedure as per [45]. A definition of mental imagery was discussed with participants and they were trained in generating mental imagery from a field (first person) perspective using a guided imagery exercise and cue cards. For each training stimulus participants were prompted to indicate mental imagery vividness on a scale from 1 to 5.

#### Picture-word cue mental imagery generation task

**Fig. 2:**
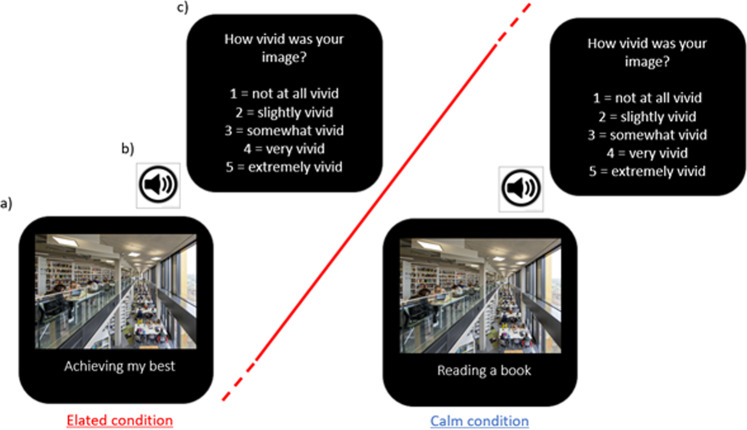
Task. Participants completed 90 trials of a picture-word cue imagery generation task. The set of pictures was identical across group and condition, but the caption differed depending on condition assignment (elated, left; calm, right).

Participants completed four neutral or mildly positive imagery practice trials of the computerised picture-word cue imagery generation task followed by 90 ‘elated’ or ‘calm’ imagery trials according to condition assignment. Each trial consisted of a picture paired with a word or phrase. Participants were asked to look at the picture, read the word or phrase, and then close their eyes and generate a mental image which combined both the picture and the word(s). Each trial consisted of a picture paired with a word or phrase that was designed, when combined to generate a mental image, to result in a positive resolution. All participants saw the same pictures, but the disambiguating word cue altered the emotional resolution dependent upon condition (Fig. 2). In the elated condition, all picture-word cues suggested an exciting positive emotional state, whereas in the calm condition, picture-word cues had calm, relaxing or more emotionally neutral resolution. For example, a photo of the university library was paired with the phrase ‘achieving my best’ in the elated condition and ‘reading a book’ in the calm condition. Stimuli were presented using E-Prime software in blocks of 30. Each picture-word cue was presented for 4500 ms (Fig. 2a) and followed by a 1000 ms auditory tone (Fig. 2b). On hearing the tone participants opened their eyes and rated vividness (Fig. 2c). Prior to starting the computerized imagery task, and again after each of the three imagery blocks, participants completed the affect measurement (see below). The procedure lasted approximately 45 minutes, with every block lasting 10–15 min. After every 10 stimuli during the imagery task, the experimenter spoke to participants, providing reinforcement and reminders for task adherence (e.g., use of field perspective, staying in the present moment in their imagery, focusing on imagery rather than verbal thought) [45, 58]. For further stimulus details see Supplementary Material.

#### Vividness ratings

After imagining each scenario, participants were asked to rate mental imagery vividness on a scale from 1 (‘not at all vivid’), to 5 (‘extremely vivid’). As in prior studies [38, 59], the rating was used to gather data as well as to encourage compliance.

#### In-task affect measurement

Participants rated their current mood prior to, at two time-points during, and after the picture-word cue imagery generation task (see above). Four affect measurement time-points were selected as a trade-off between increasing temporal resolution compared to prior studies, versus preserving participant engagement by minimizing repetition and participant burden. We included six scales of the Positive and Negative Affective Schedule Expanded form (PANAS-X; [60, 61]) and expanded it with additional BPDS-relevant mood words (see below). We refer to this expanded affect measure as the PANAS+. For each mood descriptor word (e.g., cheerful, afraid) participants indicated “to what extent [they] feel this way right now” on a 5-point scale (1, not at all to 5, extremely). The measure consisted of 34 mood adjectives across the following subscales of the PANAS-X: General Positive Affect, General Negative Affect, Joviality, Serenity, Self-Assurance, and Attentiveness. The additional seven adjectives aimed at increasing sensitivity to ‘hypomanic-like’ and unstable mood states were: ‘dynamic’ and ‘efficient’ from the Behavioural Activation for Depression Scale (BADS; [62]) to capture affect relating to increase in goal-directed behaviour; ‘unstable’, ‘impatient’ and ‘self-possessed (reverse scored)’ from the Affect Lability Scale (ALS; [63]) to assess key emotions in unstable and mixed states [4] and irritability [64]; and ‘assertive’ and ‘elated’ from the Big-5/Extraversion scale [65] and a recent bipolar mood monitoring study [66], as they are reported to be indicators of hypomanic risk [67].

#### Valence ratings of picture pleasantness

See Supplementary Material.

#### Additional measures

At the end of the experimental session, participants completed questionnaires about their subjective experiences of the mental imagery task and demand characteristics. See Supplementary Material.

### Analysis

#### Sample size calculation

We determined *post hoc* an effect size from the between-groups comparison of PANAS + total score using unequal samples, for the high (*n* = 31) and low (*n* = 30) MDQ groups. For known t-values and sample sizes available, using the formula from [68], there is a medium to large effect size (Cohen’s d) on expected differences between high vs. low MDQ groups.

#### Baseline descriptives

Demographic and baseline affect self-report variables comparing low vs. high MDQ groups were analysed using independent t-tests, or chi-square tests for categorical variables.

#### Mixed effect model analyses of affect across group, condition, and time

We used linear mixed effect model analysis to investigate changes in participant affect scores over time, and whether any such changes differed as a function of participant group and imagery condition (Hypothesis 1). Participant was modelled as a random effect while group and condition were fixed effects. Subsequently we explored the time series structure in each group and condition, using model comparisons and likelihood ratio (LR) tests to test for differences [69]. Initial linear mixed effects model analysis was conducted using all 41 PANAS+ affect words. Subsequently we analysed dissociable effects of time and condition in each group on distinct affect subtypes (Hypothesis 2). Affect subtypes were identified by conducting a hierarchical clustering analysis on the affect words of the PANAS+. Analysis was completed in R [70].

#### Analysis for moderating effect of vividness

See Supplementary Material.

## Results

### Demographic information, pre-screening and baseline affect questionnaires

High vs. low MDQ groups did not differ on age (M = 20.52, SD = 1.80; mean difference = 0.2, 95% CI [−0.71, +1.14]; t (59) = 0.46, *p* = 0.42), gender (χ^2^ (2) = 1.86, *p* = 0.39), ethnicity (white vs. other groups combined; χ^2^ (1) = 0.67, *p* = 0.41), or occupation χ^2^ (1) = 0.38, *p* = 0.53. Groups did not differ based on psychiatric screening using the MINI (Lifetime Mental Disorders χ^2^ (1) = 2.76, *p* = 0.09; Anxiety Disorders χ^2^ (1) = 0.67, *p* = 0.41; Substance Use Disorder χ^2^ (1) = 0.12, *p* = 0.72; Depressive Episode Lifetime χ^2^ (1) = 2.5, *p* = 0.11; see Table 1). Participant groups did not differ in recent depressive and anxiety symptom scores (BDI-II; t (59) = −0.35, *p* = 0.72; STAI-T; t (59) = −1.57, *p* = 0.12), or in spontaneous use of imagery (SUIS; t (59) = −1.55, *p* = 0.12). Groups showed distinct patterns of self-reported affective instability (see Supplementary Materials).

### Mixed effect model analysis of total affect score across group, condition, and time

#### Mixed effect model analysis 1 (H1: Stimulus specificity)

To begin to understand variation in affect attributable to participant, group, condition, and time, we undertook a mixed effect model analysis using total PANAS+ scores (all 41 items). This showed a positive, linear effect of time (*t* = 6.89, *p* < 0.001) through an autocorrelated (AR(1)) error structure effect and a significant group by condition interaction on total PANAS+ score (*t* = 2.736, *p* = 0.008). The main effect of time indicates affect increases as time proceeds. The group by condition interaction indicates that changes in affect differ depending both on participant group (high vs. low MDQ) and imagery condition (calm vs. elated), potentially consistent with H1. The strong random effect of participant on intercept (SD = 13.77) suggests considerable inter-participant variability. The intercept (PANAS+ score) is significantly different from zero (*t* = 20.14, *p* < 0.001).

#### Time-series structure

To investigate further the temporal structure with respect to participant, group, and condition, including to evaluate whether the direction of effects is consistent with H1, we separated the data into four sets according to group and condition (MDQ group: low [G0], high [G1]; imagery condition: calm [C0], elated [C1]) to explore the time series structure in each of these four datasets. Model comparisons and likelihood ratio tests (LRT) for the individual group/condition level showed no statistical support for correlated error structures: Within each group/condition, each affect score is independent of affect score on the previous time point (G1/C1 LRT = 1.577, *p* = 0.2092; G1/C0 LRT = 3.235, *p* = 0.0716; G0/C1 LRT = 0.521, *p* = 0.4706; G0/C0 LRT = 0.1179, *p* = 0.7313). The non-linear effects of time vary between groups.

*G0*. For low MDQ participants there is some evidence that non-linear time effects are important (G0/C0 LRT = 3.774, *p* = 0.0521; G0/C1 LRT = 3.938 *p* = 0.0472). The non-linear pattern in G0 over time depends on condition; for C1 (low MDQ/elated), the non-linear pattern increases but with decreasing amounts i.e., *decelerating*. For C0 (low MDQ/calm) the non-linear fit is not significant due to high between-participant heterogeneity (high random effect SD) (Table 2). That is, low MDQ participants experience diminishing increases in total affect score over time during elated imagery, with no evidence for affect change during calm imagery.

**Table 2:** Fixed and random effects for each group/condition using random effects model: PANAS SCORE~time, ~1|participant. Table reports intercepts and slopes for fixed effects of time (with standard errors) and the standard deviation (sd) associated with the random effects around the intercept.

Treatment	Fixed effects	Random effects
Group 1/Condition 1	Intercept: 79.09 4.51 Slope: 5.04 1.04	Intercept (sd): 14.01
Group 1/Condition 0	Intercept: 67.60 3.87 Slope: 3.26 0.88	Intercept (sd): 11.71
Group 0/Condition 1	Intercept: 70.56 3.71 Slope: 4.36 0.851	Intercept (sd): 11.52
Group 0/Condition 0	Intercept: 76.70 5.58 Slope: 3.98 1.10	Intercept (sd): 18.17

**Fig. 3:**
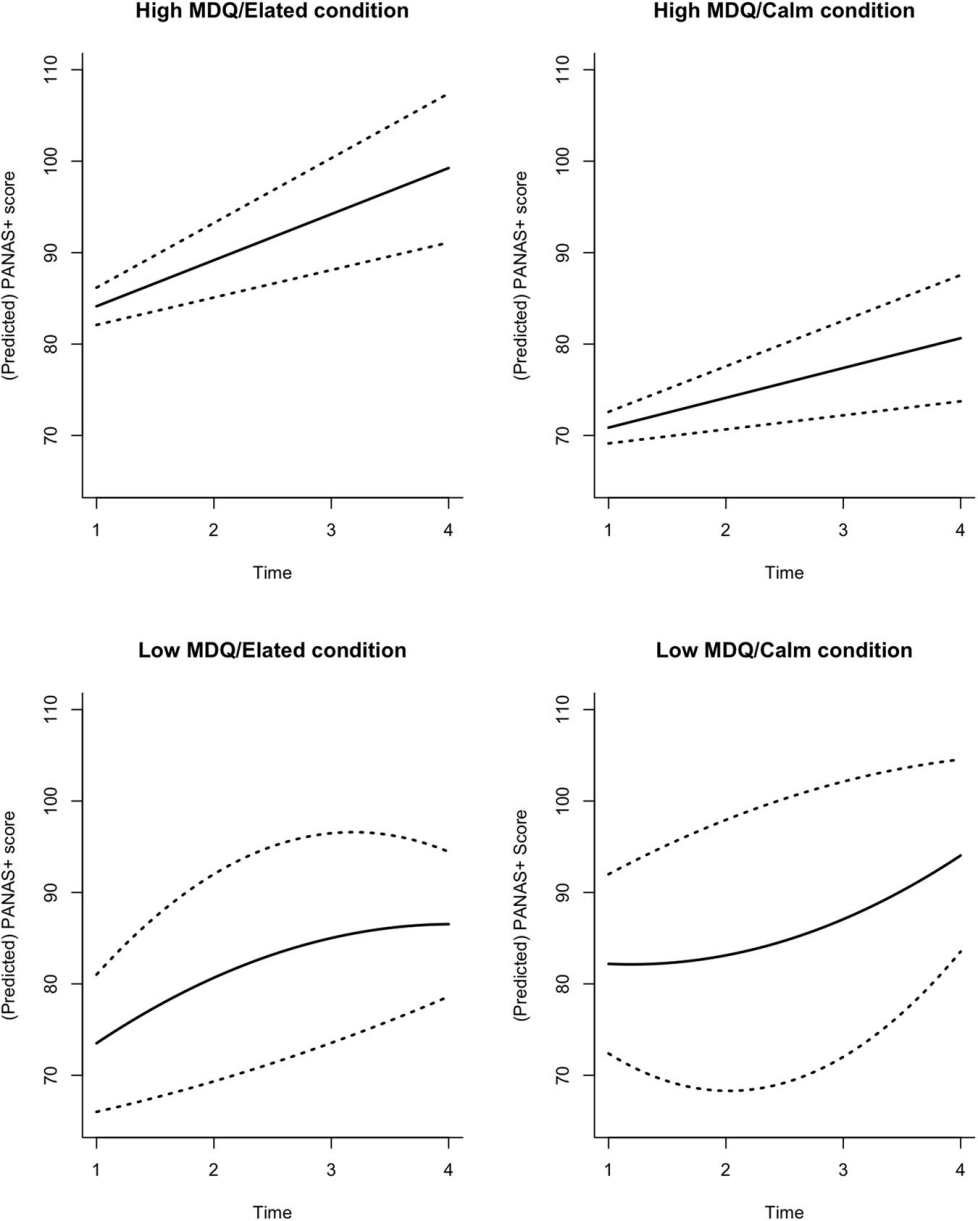
Predicted relationship from the mixed model analysis for PANAS+ affect score and time for each participant group. Participants in the high MDQ group are predicted to have additive increases in PANAS+ affect score through time, irrespective of imagery condition, with this increase steeper in the elated than calm imagery condition. Participants in the low MDQ group are predicted to have multiplicative increases in PANAS+ affect score through time, with this change significant in the elated condition only (decelerating). Solid line shows predicted relationship from linear or linear mixed model analysis; dashed line 95% confidence intervals.

*G1*. For G1 participants (high MDQ) there is no evidence for non-linear time effects comparing a quadratic and linear model with time as an explanatory variable (G1/C1 LRT = 2.246, *p* = 0.1168; G1/C0 LRT = 2.292, *p* = 0.13). Instead, for participants in the high MDQ group, affect increases additively over time (i.e., in a constant sustained manner). Furthermore, as shown by the differences in slope, affect increases faster for participants under C1 (elated) than those under C0 (calm) (Fig. 3; see also Supplementary Fig. 1 and Supplementary Table 1). That is, high MDQ participants experience sustained increases in total affect score over time during both conditions of the imagery task (i.e., in a quasi-escalation like manner), with a steeper slope in the elated compared to the calm imagery condition.

To investigate the random effects of variability between participants (within each group/condition), Table 2 summarizes the fixed and random effects for model where time is a linear explanatory variable of affect score and participant is included as a random effect. There is greatest heterogeneity amongst participants in G0/C0 (low MDQ/calm) and least amongst participants in G0/C1 (low MDQ/elated).

### Mixed effect model analysis of affect subtype scores across group, condition, and time

#### Cluster analysis

Hierarchical clustering of PANAS+ scores (all 41 words) at the first measurement time-point was used to identify affect subtype clusters (‘negative’, ‘calm-positive’, ‘active-positive’) in each group resulting in dependent variables for mixed effect model analysis 2. See Supplementary Material for cluster analysis results.

### Mixed effect model analysis 2 (H2: affect specificity)

#### Negative affect

Linear mixed effect model analysis showed no effect of time or condition on the negative affect cluster in either group. G0. In the low MDQ group there was no effect of time (*t* = 1.19, *p* = 0.238) or condition (*t* = 0.724, *p* = 0.470). The intercept differed from zero (*t* = 20.78, *p* < 0.001) and showed a modest random effect of participant (SD = 2.11). G1. In the high MDQ group there was no effect of time (*t* = 0.292, *p* = 0.771) or condition (*t* = 0.032, *p* = 0.974) on scores in the negative affect cluster. Here, the intercept differed from zero (*t* = 12.50, *p* < 0.001) with no random effect of participant (SD < 0.001). Therefore, we find no evidence that negative affect in particular is altered by our manipulation.

#### Positive affect subtypes – Low MDQ

Analysis of the two positive affect clusters in the low MDQ group showed a negative, linear effect of time (*t* = −4.42, *p* < 0.001) through an autocorrelated (AR(1)) error structure effect, that differed according to affect cluster (*t* = 9.23, *p* < 0.001) but not condition (*t* = 1.36, *p* = 0.185; see Supplementary Fig. 3). This indicates an overall modest decrease in positive affect over time that is consistent across conditions and greater for the calm-positive than the active-positive cluster. The random effect of participant on intercept (SD = 5.91) suggests moderately high inter-participant variability. The intercept is significantly different from zero (*t* = 16.02, *p* < 0.001).

#### Positive affect subtypes–High MDQ

Analysis of the two positive affect clusters in the high MDQ group showed a distinctly different pattern (Supplementary Fig. 4). Here, there was no linear effect of time (*t* = 1.00, *p* = 0.317), but instead a cluster-by-condition interaction (*t* = 5.64, *p* < 0.001). This indicates a greater than linear difference between the two positive affect clusters (‘calm-positive’, ‘active-positive’). Critically, the highest scores were found in the combination of active-positive affect cluster and elated imagery condition (C1). The random effect of participant on intercept (SD = 3.82) suggests modest inter-participant variability. The intercept is significantly different from zero (*t* = 13.05, *p* < 0.001).

#### Vividness

See Supplementary Material.

#### Valence ratings

See Supplementary Material.

#### Subjective experience and demand measures

See Supplementary Material.

## Discussion

### Overview of findings

This study took an experimental psychopathology approach to investigate positive mood amplification associated with the bipolar disorder spectrum (BPDS). We created a picture-word cue mental imagery generation task [38, 39], and used this to successfully model positive mood amplification in our subclinical BPDS-relevant sample [45]. Future work should extend these findings to a BPSD high-risk sample [71]. Here, we found that, for participants reporting high hypomanic-like experiences (high MDQ group), affect increased steeply and in a sustained manner over time (i.e., every 10–15 min, see Measures) during the positive imagery generation task. This increase was steeper in response to stimuli designed to elicit ‘elated’ mental imagery featuring (hypo)mania-related content (e.g., approach behaviour, reward-pursuit, excitement), and was markedly shallow in the ‘calm’ mental imagery comparison condition. In contrast, participants scoring low on the MDQ did not show sustained mood amplification. Furthermore, in the high MDQ group, mood amplification in the elated condition was most pronounced specifically for an active-positive affect subtype. Together, these results suggest that the magnitude and nature of BPSD mood amplification is amenable to experimental manipulation, through altering the type or content of mental imagery generated.

### Mental imagery amplifies mood dependent on MDQ group and positive imagery condition [H1]

Prior research indicates that mental imagery is vivid and emotionally evocative across the bipolar spectrum and at-risk groups, including young adults scoring highly on the MDQ [26, 40–44, 72]. In a prior study, we showed that generating vivid mental imagery in response to generically positive picture-word cues amplified mood more strongly in high (vs. medium and low) MDQ young adults [45]. However, the experience of imagery in BPDS is thought not to be ‘generic’ but particular (e.g., compelling, future-oriented; [41]). In our study, the high MDQ group’s steeper increase in affect over time in the elated vs. calm imagery condition suggests that all positive mental images are ‘not equal’ in terms of their risk for mood amplification in BPSD [73]. Generating elated, approach-related imagery leads to sustained mood amplification in a quasi-escalation like manner in high MDQs, whereas the low MDQ group experienced a decelerating pattern of mood increase, such that their initial mood increase levelled off. In turn, high MDQ mood amplification by calm imagery was markedly shallow; in other words, mood remained more stable. Therefore, in providing empirical evidence for the Emotional Amplifier Theory as applied to hypomanic-like mood amplification [26], we further show that the magnitude of this amplification varies depending on the type or content of mental imagery. This is of clear therapeutic interest.

Based on our results, future investigations should test whether positive, calm imagery may be employed (1) to modulate the degree of positive mood amplification in (hypo)manic states, and (2) to improve positive affect and thereby reduce depressive affect in BPDS in a way that helps minimize the risk of positive mood switch/amplification. Previous research has shown that time-series analysis is key to capturing mood instability over days/weeks in BPDS [41, 74, 75]. Here we demonstrate for the first time the potential of employing this approach to understanding affect change in BPDS at a micro-level, i.e., within an experimental session. This approach is also in keeping with other models highlighting that the chronometry of approach motivation system responses may explain variability of affect subtypes in BPDS [76]. Overall, our findings shed a unique light on aetiologic cognitive mechanisms of positive mood amplification in BPDS and inform research into developing better psychological prevention and treatment strategies. One caveat is that the ‘hypomanic-like’ and unstable affect words added to the PANAS + require validation.

### Differential amplification effects on distinct affect subtypes [H2]

Given our finding that mood amplification is dependent on hypomanic-like experiences and category of imagery stimuli, we proceeded to explore the impact across categories of affective response. Following an exploratory, data-driven approach, cluster analysis of affect scores revealed the expected major divide between positive and negative affect [77], as well as two positive affect subtypes: one comprising active mood states (e.g., elated, excited, energetic; ‘active-positive’ cluster), the other comprising alertly calm affect mood states (e.g., relaxed, at ease, interested; ‘calm positive’ cluster; see Supplementary Material). Our positive mental imagery task did not have any impact on the negative affect cluster, but only an impact on positive affect. This partly extends and also differs from our previous study [45], in which a positive imagery task amplified combined positive and negative affect on the PANAS-X, although imagery vividness was specifically relevant to amplifying positive affect only. The discrepancy may be secondary to refining the imagery task stimuli such that the picture-word combinations are less prone to subjective interpretation and led more directly to affective states consistent with excitement/approach readiness or contentment. Another explanation might be that the high (vs low) MDQ sample in our previous study presented with a more significant past history of anxiety and other psychiatric comorbidities compared to this study. We speculate that these clinical differences may have played a role in the modulation of positive and negative affect via positive imagery generation. What is critical however from a translational perspective, is the relevance for positive affect.

Interestingly, the impact of imagery on positive affect subtypes differed markedly according to the presence of hypomanic-like experiences. In the high MDQ group, maximum affect scores occurred in the active-positive cluster in the elated imagery condition, with lower scores in the calm condition. Hence, we suggest that, in line with our hypothesis, the elated imagery condition exerts a targeted impact on approach-related, hypomanic-relevant mood in high MDQ participants. This is congruent with evidence for intense reward and achievement-focused positive emotions in BPSD [48], with potential functional consequences for cognition, action tendency, creativity, risk-taking and wellbeing [19, 46, 47, 78, 79]. Here, we show that the magnitude of these approach-motivated emotions, while potentially a characteristic response tendency of this participant group, can be manipulated experimentally based on the category of mental imagery stimuli. It also indicates potential validity of our procedure as an experimental model of BPDS (hypo)manic mood escalation. By contrast, in the low MDQ group, we observed a steady decline in overall positive mood across both imagery conditions that was most pronounced for calm (vs. active) positive mood, consistent with a non-specific mechanism (e.g., fatigue).

### Mechanisms, clinical implications and suggestions for future research

Our findings shed new light on cognitive mechanisms of mood amplification in BPDS and suggest a number of potential implications. We show a specific mood amplification effect on a targeted population. That is, in participants scoring highly on hypomanic-like experiences, generating ‘elated’ mental imagery drives strong, sustained mood amplification, whereas, for ‘calm’ mental imagery the degree of amplification, while still sustained, is shallower. These findings are in line with existing studies on dysregulation of positive emotion in BPDS following exposure to elated visual stimuli, e.g., film clips [48, 80, 81]. Our results extend these prior findings using external stimuli to imagery self-generated in response to experimental cues. This paradigm may help efforts to model the escalation of mood due to imagination and fantasy rather than outside perceptual cues, for positive and negative affect [].

Future research should explore the link between our findings and potential functional consequences. For example, our calm vs. elated positive imagery stimuli could be used to explore the association between imagery, creativity (e.g., divergent thinking), and approach-motivation [46, 47, 79, 82, 83]. In doing so, future studies could help to understand how individuals with BPDS can be ‘touched by fire’, and crucially, when and why they ‘get burnt’ [82, 84]. It would be interesting to examine clinical BPDS samples including through periods of euthymic vs. (hypo)manic mood to determine whether the current BPDS-relevant subclinical findings extend, whether they apply across mood periods, and therefore whether imagery-based interventions are best applied to prevent vs. treat bipolar mood amplification.

Research on modifiable mechanisms of positive mood escalation can be harnessed in developing psychological interventions for BPDS. Young people presenting with hypomania are a critically underserved population and there is an urgent need to offer support beyond psycho-education and pharmacological approaches. Critically, we suggest that the current results illuminate an intervention strategy that would seek to identify, modify and dampen elated mental imagery whilst preserving or even promoting calm imagery. Interventions utilizing such strategies could be well-tolerated by patients as they could enable retention of some aspects of positive emotionality (i.e., calm, contentment, self-soothing), while potentially reducing the risk of escalation (i.e., elation). Lived-experience perspectives highlight that ambivalence towards hypomanic states is common [85], and we propose that our approach may address this and promote self-empowerment.

We suggest that combined with methods for identifying periods in which an individual may be at increased risk of mood amplification (e.g., monitoring mood, activity, and life events; cf. [74, 75]), creating targeted mental imagery interventions could provide a clinician- or self-administered psychological tool to ‘flatten the curve’ of maladaptive mood amplification while sustaining beneficial positive mood experiences.

## Supplementary information

Supplementary material

## Data Availability

The study was approved by the University of Birmingham Science, Technology, Engineering and Mathematics Ethical Review Committee (ERN 15-1435). The datasets generated and analyzed during the current study are not publicly available to protect the privacy of participants but are available from the corresponding author on reasonable request.
