# Supplementary material for: Positive moods are all alike? Differential affect amplification effects of ‘elated’ versus ‘calm’ mental imagery in young adults reporting hypomanic-like experiences
Source: Transl Psychiatry. 2022 Oct 19;12:453. doi: 10.1038/s41398-022-02213-4 (PMC9581908; doi:10.1038/s41398-022-02213-4)
Supplement: Supplementary file 1 — Supplementary material [file 41398_2022_2213_MOESM1_ESM.docx]

Supplementary Material

**1. Supplementary methods**

**Psychiatric screening.** Psychiatric screening was conducted using the Mini International Neuro-psychiatric Interview for DSM-5 (MINI; Lecrubier et al., 1997), a brief structured interview for assessing current and lifetime major psychiatric disorders in DSM-5 and ICD-10 (American Psychiatric Association, 2013; World Health Organization, 1992).

Before administration of the MINI, two questions were asked to assess history or presence of bipolar symptoms: 1) have you ever been diagnosed with any psychological distress or mental illness? If yes, do you know what? Exclusion from the study would follow if the answer were bipolar disorder I or II; 2) have you ever received any support for psychological distress or mental illness? If yes, what? Participants were excluded if they reported having taken or currently taking mood stabilizers. Psychiatric exclusion criteria based on the MINI were as follows: meeting criteria for either lifetime bipolar disorder I and II (since experimentally escalating positive mood may be harmful for these participants), or one hypomanic episode which caused “severe problem” as reported on the MDQ; meeting criteria for current depressive episode, experiencing frequent and moderate (or more) suicidal thoughts or psychotic symptoms (as these participants may have reduced ability to generate positive mental images). To verify the irrational content of psychotic ideas and major consequences, two more questions were asked with exclusion following if the participant answered YES to both: 1) Do you believe 100% in this? 2) Have those thoughts or experiences made you do something that had a major impact on your life, or has it stopped you from doing something?

**Demographic information, psychiatric screening, and baseline affect questionnaires.** The demographic information questionnaire asked participants to report on their gender identity, age, marital status, current education status (student or non-student) and ethnicity.

Section A of the Mood Disorder Questionnaire (MDQ; Hirschfeld et al., 2000) lists 13 examples of hypomanic-like experiences (e.g. ‘you had much more energy than usual’). For each item participants indicate (yes, no) whether they experienced it during a period of time in which they were ‘not their usual self’. This measure captures between-participant variability in hypomanic-like experiences (Rock et al., 2013) and has previously been used in young adult samples (Miller et al., 2011).

The Spontaneous Use of Imagery Scale (SUIS; Reisberg et al., 2003) is a self-report questionnaire measuring the tendency to use mental imagery in everyday life. For each of 12 items (e.g., ‘When I think about a series of errands I must do, I visualize the stores I will visit’) participants rate how appropriate the statement is to them (from 1, never to 5, always). The SUIS has been found to show good internal consistency (α = 0.98) and good convergent validity (Nelis et al., 2014).

The Beck Depression Inventory-Second Edition (Beck et al., 1996) measures current depressive symptomatology over the preceding two weeks.

The State Trait Anxiety Index-Trait (STAI-T; Spielberger et al., 1983) assesses trait anxiety.

The Affective Lability Scale, Short form (ALS-SF; Oliver & Simons, 2004) measures the self-reported temperamental tendency to change rapidly from euthymic mood to more extreme emotional states including elation, depression and anger. It has good reliability and high internal consistency (r =0.94) (Oliver & Simons, 2004), including in the bipolar population, with Cronbach’s α ranging 0.77–0.88 (Aas et al., 2015).

The Affective Control Scale (ACS; Williams et al., 1997) assesses regulation difficulties (i.e. fear of losing control) across a range of affective states. It has very good internal consistency and test-retest reliability (α= .94), discriminant (r=–0.17) and convergent (r =–0.72) validity.

The Affective Intensity Measure (AIM; Larsen et al., 1986) assesses self-reported strength of affective responses to typical life situations, with good internal consistency (α=0.90–0.94), test-retest reliability and construct validity. Together, these three affective measures (ALS, ACS and AIM) have been recommended to characterise affective instability across a number of populations (Marwaha et al., 2014), and are widely used in BPSD samples (Aas et al., 2015; Henry et al., 2008; Look et al., 2010).

**Valence ratings of picture pleasantness.** This measure was included to test for mood-congruent changes in interpretation bias (that is, greater evaluative conditioning), in line with past studies (Burnett Heyes et al., 2017; Holmes, Geddes, et al., 2008; O’Donnell et al., 2018; Pictet et al., 2011). Before and after taking part in the positive picture-word imagery generation task, participants viewed fifty photograph stimuli (i.e. images without words) selected randomly from the set of 90 from the imagery generation task, and rated the pleasantness of each photo on a scale from 1 (‘extremely unpleasant’) to 9 (‘extremely pleasant’) (Holmes, Geddes, et al., 2008; Pictet et al., 2011). Valence ratings were analyzed with a three-way mixed ANOVA with pleasantness ratings as within-subject factor and MDQ score and experimental condition as between-subject factors.

**Imagery generation task stimuli.** Picture stimuli were shown centered on the 13’’ VDU against a black background. Pictures varied within a width from 360 and 640, and a height between 338 and 453 pixels. Images comprised photos selected from previous studies (Burnett Heyes et al., 2017; O’Donnell et al., 2018), taken by the authors, or downloaded from the Internet. Words were displayed centered in white beneath the image, in Arial size 30. Stimuli were presented in a randomised order.

Stimuli in the elated condition represented the following themes relevant to (hypo)manic states and experiences: achievement (i.e. reward pursuit and competition; e.g. picture of a race with the phrase ‘almost winning’), inflation of self-esteem (e.g. picture of a class with the phrase ‘I’m so clever’), and behavioural activation (e.g. picture of a bed at night with the phrase ‘still full of energy’) (Gruber & Johnson, 2009). Stimuli in the calm condition represented the following themes representing positive emotional experiences not typically associated with (hypo)mania: rest and relaxation (e.g. picture of tea and biscuits with the phrase ‘tea break’), affiliation and cooperation (e.g. picture of a team sport with the phrase ‘team effort’), and peace and delight implying self-acceptance (Hartig et al., 2003). In addition, each condition incorporated roughly equal proportions of stimuli across each of the following dimensions: 1) self-focus: focus on the participant engaging in the scenario; 2) other-focus: focus on other people present in the scenario; 3) affective hyperesthesia: focus on an object or an event (Hardy et al., 1986).

**Additional measures.** At the end of the experimental session, participants completed a questionnaire about their subjective experience of having generated mental images. This included questions on imagery ease (how difficult they found the task; how difficult they found it to combine the picture with the word), how much they verbally analysed the picture-word combinations, and how much they used the field (first person) perspective. Participants reported their answers on scale from 1 (‘very easy/none of the time) to 9 (‘very difficult/all the time) (Pictet et al., 2011). Demand characteristics of the task were also investigated (Holmes, Mathews, et al., 2008). Participants reported whether they thought mental imagery during the task affected their positive and negative emotions on a 9-point scale (1, ‘no change in how positive/negative I felt’; 9, ‘great change in how positive/negative I felt’). Subjective experience and demand characteristics were analyzed using two-way univariate ANOVA with MDQ group (low, high) and experimental condition (calm, elated) as fixed factors.

**Analysis for moderating effect of vividness on mood amplification.** Since participants rated vividness on each trial but mood after each block of 30 trials, we computed summary measures of vividness for each block. Three summary measures were computed: Moment metric (unbiased mean), vividness covariance, and a measure encapsulating the relationship between the two. Interestingly, the covariance measure did not differ significantly from zero, indicating no autocorrelation effects on vividness. This means that imagery vividness is independent across trials and participants. As such, it is potentially a valid, accurate indication of imagery vividness on each trial that is unconfounded by individual participant response tendencies and cumulative effects across the task. We subjected the three vividness summary measures to linear mixed model analyses to pinpoint any the relationship between their change over time and relative mood change, MDQ group and imagery condition. Results are shown below in the Supplementary results section.

**Supplementary results**

**Baseline affect questionnaires.** High and low MDQ Groups showed distinct patterns of mood instability with the high MDQ group reporting greater fear of affect change (ACS; t (58) = -2.64, p = .01) and greater intensity of mood lability (AIM; t (59) = -2.37, p = .02), with no difference in self-reported mood oscillations (ALS-SF; t (59) = -.51, p = .60).

**Supplementary Figure 1.** Individual participant PANAS+ affect scores at each time point, for each group/condition combination. **
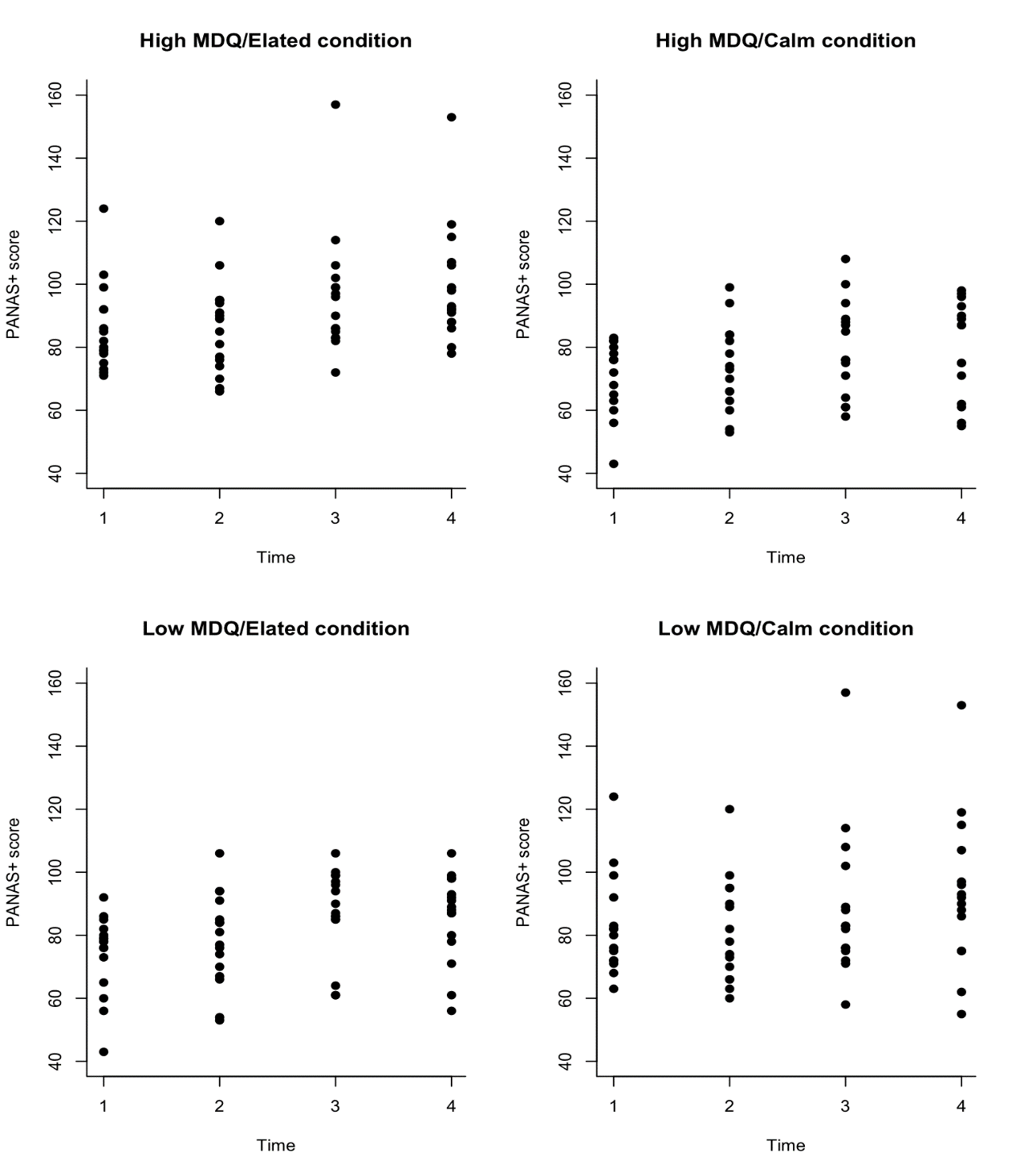
**

**Supplementary Table 1. Group and condition mean PANAS+ scores at each time point.**

| Group/condition | T=1 | T=2 | T=3 | T=4 |
| --- | --- | --- | --- | --- |
| High MDQ/Elated | 85.625 (SE=3.475) | 86.00  (SE=3.651) | 96.063  (SE=4.859) | 99.063  (SE=4.608) |
| High MDQ/Calm | 70.667 (SE=2.951) | 73.333  (SE=3.520) | 79.533  (SE=3.901) | 79.467  (SE=3.982) |
| Low MDQ/Elated | 72.866 (SE=4.452) | 69.333  (SE=4.075) | 81.333  (SE=5.074) | 79.133  (SE=5.203) |
| Low MDQ/Calm | 79.133  (SE=4.580) | 81.20  (SE=5.121) | 90.133  (SE=6.648) | 90.267  (SE=7.354) |

**Cluster analysis results.** Most relevant natural clusters from the PANAS+ word groupings identify five distinct groups of which three have a priori relevance for our analysis. These are negative, calm-positive and active-positive clusters. From this hierarchical clustering analysis, we identify two main results. The first result is that negative mood forms a distinct cluster from positive mood. While this grouping is similar, it is not identical between the low and high MDQ groups with 11 words shared out of a total of 13 across the negative clusters. The second result is that within the positive mood cluster, we identify calm and active classifications. Although this identification and, hence, the labelling is heuristically applied, it should be subjected to further investigation in future work, particularly exploring the structure of affect in different participant groups. Again, there are similarities between the low and high MDQ groups with 5 unique words shared (out of a total of 9 words) in the calm positive grouping and 9 words shared (out of a total of 15 words) in the active positive grouping. As this clustering analysis highlights differences between these MDQ groups, we performed the linear mixed effect model analysis on time and condition in each group separately.


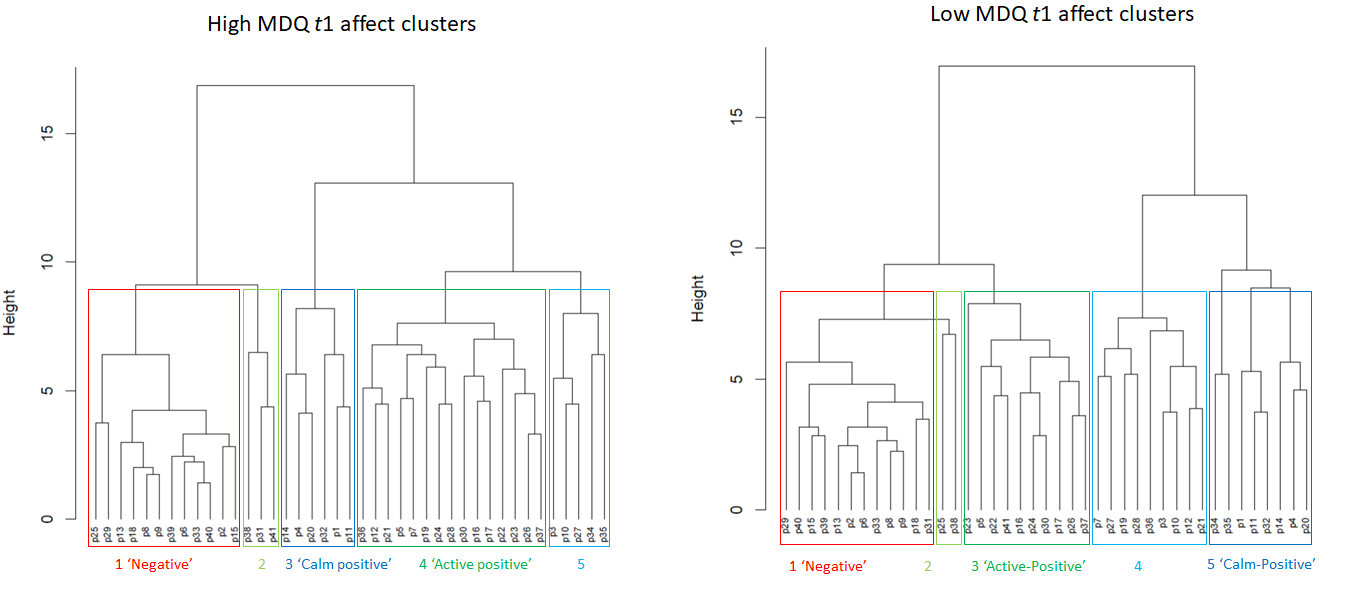


Supplementary Figure 2. Cluster analysis of PANAS+ (p) mood words at baseline

Left: High MDQ Group. Cluster analysis revealed three clear mood clusters (1, 3, 4) and two smaller clusters (2, 5). Proposed cluster labels and words included in each cluster are as follows. Cluster 1 ‘negative’. Nervous (p25), jittery (p29), hostile (p13), ashamed (p18), guilty (p8), scared (p9), impatient (p39), upset (p6), afraid (p33), unstable (p40), distressed (p2), irritable (p15). Cluster 2. Self-possessed (p38), daring (p31), dynamic (p41). Cluster 3 ‘calm positive’. Concerned (p14), interested (p4), attentive (p20), relaxed (p32), at ease (p1), calm (p11). Cluster 4 ‘active positive’. Assertive (p36), joyful (p12), lively (p21), bold (p5), strong (p7), enthusiastic (p19), energetic (p24), determined (p28), active (p30), excited (p16), proud (p17), inspired (p22), fearless (p23), delighted (p26), elated (p37). Cluster 5 – happy (p3), cheerful (p10), confident (p27), alert (p34), efficient (p35).

**Right: Low MDQ Group.** Similar but not identical, five clusters were identified: three clear and roughly comparable to the High MDQ Group (1, 3, 5) and 2 less clear clusters (2, 4). Proposed cluster labels and words included in each cluster are as follows. **Cluster 1 ‘negative’.** Jittery (p29), unstable (p40), irritable (p15), impatient (p39), hostile (p13), distressed (p2), upset (p6), afraid (p33), guilty (p8), scared (p9), ashamed (18), daring (p31). **Cluster 2**. Nervous (p25), self-possessed (p38). **Cluster 3 ‘active positive’.** Fearless (p23), bold (p5), inspired (p22), dynamic (p41), excited (p16), energetic (p24), active (p30), proud (p17), delighted (p26), elated (p37). **Cluster 4.** Strong (p7), confident (p27), enthusiastic (p19), determined (p28), assertive (p36), happy (p3), cheerful (p10), joyful (p12), lively (p21). **Cluster 5 ‘calm positive’.** Alert (p34), efficient (p35), at ease (p1), calm (p11), relaxed (p32), concentrating (p14), interested (p4), attentive (p20).

**
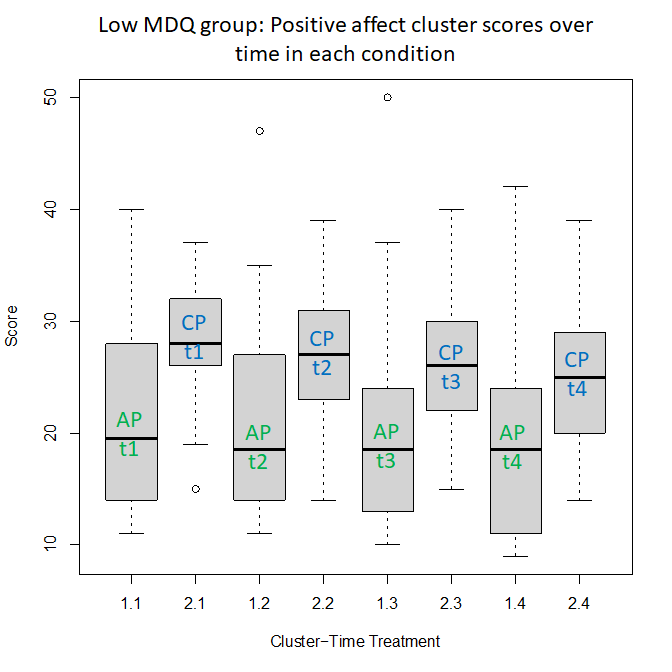
**

**Supplementary Figure 3. Boxplot showing effects of Calm-Positive and Active-Positive affect subcluster and time on affect score in the Low MDQ Group.** Linear mixed model analysis reveals significant effects of affect cluster (slope=6.615 (SE=0.717) p<0.001) and time (slope=-0.968 (SE=0.219) p<0.001) with no effect of condition (slope=-3.165 (SE=2.329), p=0.185). Intercept is significantly different from zero (intercept= 24.078 (SE=1.813), p<0.001). x-axis increments as follows: Cluster: 1=Calm-Positive, 2=Active-Positive; Time: 1=t1, 2=t2, 3=t3, 4=t4. Positive affect score decreases with time irrespective of imagery condition. The decrease is steeper for the Calm-Positive cluster.

**
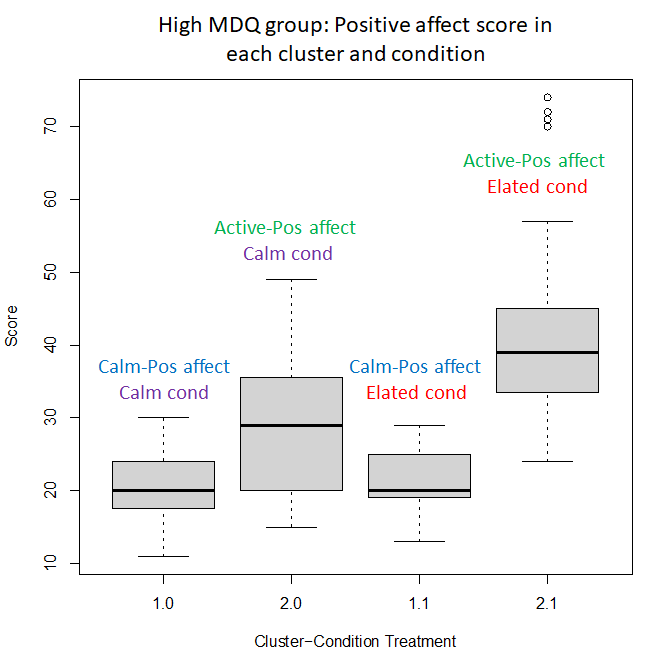
Supplementary Figure 4. Boxplot showing the effects of the Calm-Positive and Active-Positive mood subcluster and Condition on affect score in the High MDQ Group.** Linear mixed model analysis reveals a significant interaction between cluster and condition (slope=10.914 (SE=1.936) p<0.001) but no effect of time (slope= 1.039 (SE=2.201), p=0.64). Intercept is significantly different from zero (intercept=20.639 (SE=1.581), p<0.001). x-axis increments as follows: Cluster: 1=Calm-Positive, 2=Active-Positive; Condition: 0=Calm, 1=Elated. Affect score is highest for Active-Positive Affect in the Elated imagery condition.

**Vividness results.** To test the hypothesis that vividness exaggerates the impact of the experimental imagery manipulation, we used a linear mixed model to investigate how vividness changes with relative mood change (defined as the log difference between total PANAS+ score at time t and total PANAS+ score at time t-1 (log(Mt/Mt-1)), MDQ group and condition.

As vividness was determined over 30 individual trials, three different measures of vividness were computed to deal with autocorrelation effects in vividness: (i) a moment matric (based on the mean, variance and skewness in vividness for each participant over the 30 trials within a mood trial; (ii) the AR(1) covariance in vividness for each participant over the 30 trials within a mood trial and (iii) the dominant eigenvalue in the AR(1) variance-covariance matrix.

There were no statistically significant effects of mood change, MDQ or condition on vividness across the three mood trials (for any of the vividness measures). These results are summarized in the tables below.

**Supplementary Table 2. Vividness Measure: Moments**

|  | Intercept | | Mood Change | | MDQ Group | | Condition | |
| --- | --- | --- | --- | --- | --- | --- | --- | --- |
|  | F-value | p-value | F-value | p-value | F-value | p-value | F-value | p-value |
| Time 1 | 3080.923 | <0.001 | 0.196 | 0.659 | 0.571 | 0.452 | 1.353 | 0.250 |
| Time 2 | 3491.310 | <0.001 | 1.373 | 0.246 | 0.074 | 0.786 | 0.557 | 0.458 |
| Time 3 | 3342.669 | <0.001 | 2.275 | 0.137 | 2.995 | 0.089 | 0.093 | 0.762 |

Vividness moment metric is significantly different from zero, with no effect of mood change, MDQ group or condition on this metric.

**Supplementary Table 3. Vividness Measure: AR(1) Covariance**

|  | Intercept | | Mood Change | | MDQ Group | | Condition | |
| --- | --- | --- | --- | --- | --- | --- | --- | --- |
|  | F-value | p-value | F-value | p-value | F-value | p-value | F-value | p-value |
| Time 1 | 0.207 | 0.658 | 0.308 | 0.581 | 0.467 | 0.497 | 0.03 | 0.854 |
| Time 2 | 1.044 | 0.311 | 0.232 | 0.632 | 0.004 | 0.946 | 0.538 | 0.466 |
| Time 3 | 0.788 | 0.379 | 0.001 | 0.970 | 0.006 | 0.771 | 0.303 | 0.584 |

Vividness covariance is not significantly different from zero, indicating no significant autocorrelative effect (i.e. vividness ratings at each trial are independent), with no effect of mood change, MDQ group or condition on this metric.

**Supplementary Table 4. Vividness Measure: AR(1) Var-Covar Matrix**

|  | Intercept | | Mood Change | | MDQ Group | | Condition | |
| --- | --- | --- | --- | --- | --- | --- | --- | --- |
|  | F-value | p-value | F-value | p-value | F-value | p-value | F-value | p-value |
| Time 1 | 431.707 | <0.001 | 0.913 | 0.343 | 0.312 | 0.578 | 0.968 | 0.329 |
| Time 2 | 175.653 | <0.001 | 1.059 | 0.308 | 0.105 | 0.747 | 1.238 | 0.271 |
| Time 3 | 184.466 | <0.001 | 4.659 | 0.0351 | 0.02 | 0.889 | 0.703 | 0.405 |

Dominant eigenvalue of t the AR(1)-V-C matrix is significantly different from zero (expected as this is the principal axis of variance). No significant effect of mood change at time point 1 or 2. The significant effect of mood change on time point 3 using the AR(1) V-C metric is a result of a high mood score associated with a single participant. No effect of MDQ group or condition on this metric of vividness.

**Valence ratings of picture pleasantness.** A three-way mixed ANOVA on valence ratings from pre- (M = 5.99, SD = .08) to post- (M = 6.29, SD = .09) the imagery generation task showed a significant increase in picture pleasantness (F (1,55) = 19.7, p <.001) across participants, with no effect of MDQ group (F (1,55) = .99, p =.32), condition (F (1, 55) = .11 p = .74) or their interaction (F (1,55) = .19, p = .66), consistent with an increase in positive interpretation bias.

**Subjective experience and demand measures.** Overall, participants did not report difficulty in generating mental images (M = 3.3, SD = 1,5), in combining the picture with the word-cue (M = 3.3, SD = 1.5) and in adopting field imagery (M = 7.6, SD = 1.7) without verbally analysing (M = 3.9, SD= 2.3). Furthermore, participant groups did not differ on these variables (see Manuscript, Table 2). There was no group difference in participants’ subjective perception that their negative mood had changed after the imagery task (M = 3.3, SD = 2.2). Groups did however differ in their subjective perception that their positive mood had changed, with the high MDQ group reporting that they felt more positive after the imagery task (low MDQ, M = 5.3, SD =.39; high MDQ, M = 6.4, SD = .33) regardless of the imagery condition to which they had been assigned (see Supplementary Table 5, below).

**Supplementary Table 5.** Results of two-way univariate ANOVA on Subjective Experience and Demand Characteristics

| **Two-way univariate ANOVA** | **Interaction** | | **MDQ** | | **Condition** | |
| --- | --- | --- | --- | --- | --- | --- |
|  | F | p-value | F | p-value | F | p-value |
| **Generate Mental Images** | .02 | .88 | .02 | .88 | .09 | .75 |
| **Combine Picture Word-cue** | .03 | .87 | .03 | .87 | .46 | .5 |
| **Use of Field Perspective** | .74 | .39 | .35 | .56 | .04 | .83 |
| **Verbally Analyse** | .56 | .46 | .17 | .68 | .4 | .53 |
| **Change of Positive Emotions** | .83 | .37 | 4.4 | .04* | 2.5 | .11 |

**Post hoc analysis of correspondence between mood change and subjective experience of mood change in the high MDQ group.** Above, we found that self-reported (i.e. subjective) change in positive mood was greater in the high vs low MDQ group, with no effect of condition. We conducted a post hoc analysis to explore the basis of this effect in the high MDQ group. We conducted a Spearman’s rank correlation between high MDQs’ subjective positivity scores and change in PANAS+ positive mood score from time point 1 to time point 4, collapsed across conditions. In other words, this analysis tests whether individual variation in high MDQ participants’ overall sense of whether their mood became more positive corresponded to individual variation in positive mood score change. We conducted this analysis separately for previously validated PANAS-X positive mood words versus the ‘hypomanic-like’ positive mood words added for the current study.

The Spearman's rank correlation between subjective demand score and difference in total score for standard positive words was 0.217. However, this sample correlation is not significantly different from zero (t=1.196 on 29df p=0.121; from a resampling distribution: 2.5% quantile= -0.145, 97.5% quantile = 0.546). Similarly, the Spearman's rank correlation between subjective demand score and difference in total PANAS score for unique positive words is 0.243. Again, this sample correlation is not significantly different from zero (t=1.349 on 29df p=0.094; from a resampling distribution: 2.5% quantile= -0.154, 97.5% quantile = 0.535).

**Supplementary discussion**

**No effect of vividness [H3]**

Based on prior work (O’Donnell et al., 2018) we hypothesised that mood amplification across the mental imagery generation task would be dependent on imagery vividness. For each picture-word cue, participants generated mental imagery and used a numeric scale to rate imagery vividness. Since participants rated vividness on each trial but mood after each block of 30 trials, we computed summary measures of vividness for each block. Three summary measures were computed: Moment metric (unbiased mean), vividness covariance, and a measure encapsulating the relationship between the two. Interestingly, the covariance measure did not differ significantly from zero, indicating not autocorrelation effects on vividness. This means that imagery vividness is independent across trials and participants. As such, it is potentially a valid, accurate indication of imagery vividness on each trial that is unconfounded by individual participant response tendencies and cumulative effects across the task. Therefore, we recommend vividness ratings continue to be elicited in future studies, and we further recommend the summary metrics we use here.

We subjected the three vividness summary measures to linear mixed model analyses to pinpoint any the relationship between their change over time and relative mood change, MDQ group and imagery condition. Contrary to our hypothesis, we found no significant effects. That is, mood amplification did not depend on vividness ratings.

There are a number of potential explanations for this null effect. First, vividness may operate as a threshold mechanism, with a certain level of imagery vividness required to see an emotional impact but beyond this level, increasing vividness does not increase emotional impact. Since we excluded participants who reported low imagery vividness in daily life (based on the SUIS), this is conceivable in our study. Indeed, evidence suggests that imagery vividness is not amenable to improvement via training (Rademaker & Pearson, 2012), in contrast to metacognitive awareness of mental imagery (Pearson et al., 2011; Rademaker & Pearson, 2012). Second, our vividness scale had a limited range (five discrete rating points), and perhaps not enough potential variance to capture hypothesised effects, especially at the high end of the scale. Third, the scale had a dual purpose in our study. In addition to providing vividness data on every trial, the scale was used to provide a common language for discussing mental imagery and optimising its vividness. During the training phase and after every 10^th^ trial, the experimenter spoke to the participant to enquire about their images and experience of the task, giving reinforcement and reminders where needed (e.g., to use field perspective, to focus on imagery instead of verbal thoughts). Therefore, potentially, the experimental procedure produced demand characteristics on the vividness ratings (although the lack of autocorrelative effects counters this notion). Finally, the null effect of vividness could indicate that imagery specifically does not give rise to the mood amplification effects in our study, and instead the task acts more as a general mood induction procedure. However, given evidence that mental imagery accompanies both sensory perception and stimulus-independent processing, in addition to a wealth of evidence that imagery generation alters affective states across a variety of populations (Hirsch & Holmes, 2007; Hoppe et al., 2021; Kessler et al., 2018; Rachman, 2007), we do not favour this explanation. We suggest that future studies further explore the role of vividness using an expanded operating range for vividness scores and converging behavioural and neurophysiological imagery markers (Pearson et al., 2015).

**Valence ratings.** Picture pleasantness ratings increased pre/post-imagery irrespective of MDQ group and condition assignment. Results showing mood-congruent change in interpretation bias following the picture-word cue imagery generation task build on prior literature that interprets findings from the picture-word cue generation task in line with an evaluative conditioning mechanism. That is, changes in valence ratings of a stimulus result from pairing that stimulus with another positive or negative stimulus (De Houwer et al., 2001). In the prior literature, valence (pleasantness) ratings of stimuli increase or decrease in accordance with condition assignment, including positive/negative imagery valence (Holmes et al 2008 Experiment 2; Pictet et al 2011), more so for positively-valenced field vs. observer perspective imagery (Burnett Heyes et al. 2017). This suggests that in non-clinical general population samples, valence ratings are influenced by aspects of the imagery task. In the current study, we found no evidence that valence changes pre/post imagery task differ depending on MDQ group and positive imagery condition.

**Subjective experience and demand measures.** To better understand findings from mixed model analysis 1 indicating that mental imagery amplifies mood dependent on MDQ group and positive imagery condition [H1] we examined the results from analysis of scores on the additional measures, specifically whether participants felt their mood had changed as a result of the imagery task. This analysis revealed that the high (vs. low) MDQ group reported experiencing significantly greater change in positive mood after both conditions of the experimental imagery task. There are multiple potential reasons for this result. It might be that high MDQs were aware of their mood changes, showing some insight on their emotional sphere. Our post hoc analysis did not show support for this possibility, but nevertheless the effect warrants further investigation (especially given evidence that positive therapeutic outcomes are strengthened by the psychological mindedness of patients; Nyklíček et al., 2010; Piper et al., 1994). Alternatively, the increased subjective change in positive mood in the high vs. low MDQ group could reflect greater sensitivity to task demands.

**Supplementary bibliograpy**

Aas, M., Pedersen, G., Henry, C., Bjella, T., Bellivier, F., Leboyer, M., Kahn, J.-P., Cohen, R. F., Gard, S., Aminoff, S. R., Lagerberg, T. V., Andreassen, O. A., Melle, I., & Etain, B. (2015). Psychometric properties of the Affective Lability Scale (54 and 18-item version) in patients with bipolar disorder, first-degree relatives, and healthy controls. *Journal of Affective Disorders*, *172*, 375–380.

American Psychiatric Association. (2013). *Diagnostic and statistical manual of mental disorders (DSM-5)* (5^a^ ed.). American Psychiatric Pub.

Beck, A. T., Steer, R. A., & Brown, G. K. (1996). *Manual for the beck depression inventory-II*. San Antonio, TX: Psychological Corporation.

Burnett Heyes, S., Pictet, A., Mitchell, H., Raeder, S. M., Lau, J. Y. F., Holmes, E. A., & Blackwell, S. E. (2017). Mental imagery-based training to modify mood and cognitive bias in adolescents: Effects of valence and perspective. *Cognitive Therapy and Research*, *41*(1), 73–88. https://doi.org/(doi:10.1007/s10608-016-9795-8

De Houwer, J., Thomas, S., & Baeyens, F. (2001). Association learning of likes and dislikes: A review of 25 years of research on human evaluative conditioning. *Psychological Bulletin*, *127*, 853–869. https://doi.org/10.1037/0033-2909.127.6.853

Gruber, J., & Johnson, S. L. (2009). Positive emotional traits and ambitious goals among people at risk for mania: The need for specificity. *International Journal of Cognitive Therapy*, *2*(2), 176–187. https://doi.org/10.1521/ijct.2009.2.2.176

Hardy, M. C., Lancrenon, S., & Lecrubier, Y. (1986). Construction et validation d’une nouvvelle échelle biaxiale d’évaluation des états maniaques. *Psychiatry and Psychobiology*, *1*(3), 221–231. https://doi.org/10.1017/S0767399X00000079

Hartig, T., Evans, G. W., Jamner, L. D., Davis, D. S., & Gärling, T. (2003). Tracking restoration in natural and urban field settings. *Journal of Environmental Psychology*, *23*(2), 109–123. https://doi.org/10.1016/S0272-4944(02)00109-3

Henry, C., Van den Bulke, D., Bellivier, F., Roy, I., Swendsen, J., M’Baïlara, K., Siever, L. J., & Leboyer, M. (2008). Affective lability and affect intensity as core dimensions of bipolar disorders during euthymic period. *Psychiatry Research*, *159*(1–2), 1–6.

Hirsch, C. R., & Holmes, E. A. (2007). Mental imagery in anxiety disorders. *Psychiatry*, *6*(4), 161–165. https://doi.org/10.1016/j.mppsy.2007.01.005

Hirschfeld, R. M. A., Williams, J. B. W., Spitzer, R. L., Calabrese, J. R., Flynn, L., Keck, P. E., Lewis, L., McElroy, S. L., Post, R. M., Rapport, D. J., Russell, J. M., Sachs, G. S., & Zajecka, J. (2000). Development and validation of a screening instrument for bipolar spectrum disorder: The mood disorder questionnaire. *American Journal of Psychiatry*, *157*(11), 1873–1875. https://doi.org/10.1176/appi.ajp.157.11.1873

Holmes, E. A., Geddes, J. R., Colom, F., & Goodwin, G. M. (2008). Mental imagery as an emotional amplifier: Application to bipolar disorder. *Behaviour research and therapy*, *46*(12), 1251–1258. https://doi.org/10.1016/j.brat.2008.09.005

Holmes, E. A., Mathews, A., Mackintosh, B., & Dalgleish, T. (2008). The causal effect of mental imagery on emotion assessed using picture-word cues. *Emotion*, *8*(3), 395.

Hoppe, J. M., Holmes, E. A., & Agren, T. (2021). Exploring the neural basis of fear produced by mental imagery: Imaginal exposure in individuals fearful of spiders. *Philosophical Transactions of the Royal Society B*, *376*(1817), 20190690. https://doi.org/10.1098/rstb.2019.0690

Kessler, H., Holmes, E. A., Blackwell, S. E., Schmidt, A.-C., Schweer, J. M., Bücker, A., Herpertz, S., Axmacher, N., & Kehyayan, A. (2018). Reducing intrusive memories of trauma using a visuospatial interference intervention with inpatients with posttraumatic stress disorder (PTSD). *Journal of Consulting and Clinical Psychology*, *86*(12), 1076–1090.

Larsen, R., Diener, E., & Emmons, R. (1986). Affect intensity and reactions to daily life events. *Journal of personality and social psychology*, *51*(4), 803. https://doi.org/10.1037/0022-3514.51.4.803

Lecrubier, Y., Sheehan, D., Weiller, E., Amorim, P., Bonora, I., Harnett Sheehan, K., Janavs, J., & Dunbar, G. (1997). The Mini International Neuropsychiatric Interview (MINI). A short diagnostic structured interview: Reliability and validity according to the CIDI. *European Psychiatry*, *12*(5), 224–231. https://doi.org/10.1016/S0924-9338(97)83296-8

Look, A. E., Flory, J. D., Harvey, P. D., & Siever, L. J. (2010). Psychometric properties of a short form of the Affective Lability Scale (ALS-18). *Personality and Individual Differences*, *49*(3), 187–191.

Marwaha, S., He, Z., Broome, M., Singh, S. P., Scott, J., Eyden, J., & Wolke, D. (2014). How is affective instability defined and measured? A systematic review. *Psychological Medicine*, *44*(9), 1793–1808. https://doi.org/10.1017/S0033291713002407

Miller, C. J., Johnson, S. L., Kwapil, T. R., & Carver, C. S. (2011). Three studies on self-report scales to detect bipolar disorder. *Journal of Affective Disorders*, *128*(3), 199–210. https://doi.org/10.1016/j.jad.2010.07.012

Nelis, S., Holmes, E. A., Griffith, J. W., & Raes, F. (2014). Mental imagery during daily life: Psychometric evaluation of the Spontaneous Use of Imagery Scale (SUIS). *Psychologica Belgica*, *54*(1), 19–32.

Nyklíček, I., Poot, J. C., & van Opstal, J. (2010). Psychological mindedness in relation to personality and coping in a sample of young adult psychiatric patients. *Journal of clinical psychology*, *66*(1), 34–45. https://doi.org/10.1002/jclp.20627

O’Donnell, C., Di Simplicio, M., Brown, R., Holmes, E. A., & Burnett Heyes, S. (2018). The role of mental imagery in mood amplification: An investigation across subclinical features of bipolar disorders. *Cortex*, *105*, 104–117. https://doi.org/10.1016/j.cortex.2017.08.010

Oliver, M. N. I., & Simons, J. S. (2004). The affective lability scales: Development of a short-form measure. *Personality and Individual Differences*, *37*(6), 1279–1288. https://doi.org/10.1016/j.paid.2003.12.013

Pearson, J., Naselaris, T., Holmes, E. A., & Kosslyn, S. M. (2015). Mental imagery: Functional mechanisms and clinical applications. *Trends in Cognitive Sciences*, *19*(10), 590–602.

Pearson, J., Rademaker, R. L., & Tong, F. (2011). Evaluating the mind’s eye: The metacognition of visual imagery. *Psychological Science*, *22*(12), 1535–1542.

Pictet, A., Coughtrey, A. E., Mathews, A., & Holmes, E. A. (2011). Fishing for happiness: The effects of generating positive imagery on mood and behaviour. *Behaviour Research and Therapy*, *49*(12), 885–891.

Piper, W. E., Joyce, A. S., Azim, H. F., & Rosie, J. S. (1994). Patient characteristics and success in day treatment. *Journal of nervous and Mental Disease*. https://doi.org/10.1097/00005053-199407000-00003

Rachman, S. (2007). Unwanted intrusive images in obsessive compulsive disorders. *Journal of behavior therapy and experimental psychiatry*, *38*(4), 402–410. https://doi.org/10.1016/j.jbtep.2007.10.008

Rademaker, R. L., & Pearson, J. (2012). Training visual imagery: Improvements of metacognition, but not imagery strength. *Frontiers in Psychology*, *3*.

Reisberg, D., Pearson, D. G., & Kosslyn, S. M. (2003). Intuitions and introspections about imagery: The role of imagery experience in shaping an investigator’s theoretical views. *Applied Cognitive Psychology*, *17*(2), 147–160. https://doi.org/10.1002/acp.858

Rock, P. L., Chandler, R. A., Harmer, C. J., Rogers, R. D., & Goodwin, G. M. (2013). The common bipolar phenotype in young people. *International Journal of Bipolar Disorders*, *1*(1), 19. https://doi.org/10.1186/2194-7511-1-19

Spielberger, C. D., Gorsuch, R. L., Lushene, R. E., Vagg, P. R., & Jacobs, G. A. (1983). State-trait anxiety inventory. Palo Alto. *CA: Mind Garden*.

Williams, K. E., Chambless, D. L., & Ahrens, A. (1997). Are emotions frightening? An extension of the fear of fear construct. *Behaviour Research and Therapy*, *35*(3), 239–248.

World Health Organization. (1992). *The ICD-10 classification of mental and behavioural disorders: Clinical descriptions and diagnostic guidelines*. World Health Organization.
